# Multiple immunoassay interference in a patient with falsely elevated calcitonin

**DOI:** 10.20945/2359-4292-2023-0074

**Published:** 2023-12-01

**Authors:** Mehmet Cagri Unal, Aslihan Cavunt Bayraktar, Tevfik Uslu, Serkan Yener

**Affiliations:** 1 Dokuz Eylul University Faculty of Medicine Division of Endocrinology and Metabolism Izmir Turkey Dokuz Eylul University Faculty of Medicine, Division of Endocrinology and Metabolism, Izmir, Turkey; 2 Duzen Laboratories Group Ankara Turkey Duzen Laboratories Group, Ankara, Turkey; 3 Dokuz Eylul University Faculty of Medicine Endocrinology Laboratory Izmir Turkey Dokuz Eylul University Faculty of Medicine, Endocrinology Laboratory, Izmir, Turkey

## Abstract

Calcitonin (CT) is a diagnostic and follow-up marker of medullary thyroid carcinoma. Heterophile antibodies (HAbs) may interfere during immunometric assay measurements and result in falsely high CT levels and different markers. A 50-year-old female patient was referred to our institution for elevated CT levels (3,199 pg/mL [0-11,5]). Physical examination and thyroid ultrasonography show no thyroid nodules. Because of the discrepancy between the clinical picture and the laboratory results, various markers and hormones were examined to determine whether there was any interference in the immunometric assay. Thyroglobulin (Tg) and Adrenocorticotropic hormone (ACTH) levels were also found inaccurately elevated. After precipitation with polyethylene glycol, CT, Tg, and ACTH levels markedly decreased, showing macro-aggregates. Also, serial dilutions showed non-linearity in plasma concentrations. Additionally, CT samples were pretreated with a heterophilic blocking tube before measuring, and the CT level decreased to < 0.1 pg/mL, suggesting a HAb presence. Immunoassay interference should be considered when conflicting laboratory data are observed. This may help reduce the amount of unnecessary laboratory and imaging studies and prevent patients from complex diagnostic procedures.

## INTRODUCTION

Calcitonin (CT) is a polypeptide hormone that is one of the biochemical markers of medullary thyroid carcinoma (MTC). Serum CT concentrations were correlated with tumor size in patients with MTC (1). Besides, different physiological and pathologic disorders have been associated with higher CT levels, such as hypercalcemia, hypergastrinemia, and renal insufficiency (2). One of the rare causes of hypercalcitoninemia is immunoassay (IA) interference (3).

IAs are commonly used in clinical laboratories for different hormone analyses that can be classified into competitive and noncompetitive (sandwich) methods. Sandwich IAs are suitable for larger molecules, including polypeptide hormones, CT, and thyroglobulin (Tg) (4).

Heterophile antibodies (HAbs) in the serum may develop after exposure to animal or animal products that can last only a few days to months or years. HAbs may interact with IA and cause inappropriately high or rarely low values. In a sandwich IA, a HAb can create a bridge between captured and labeled antibodies, leading to false-positive interference in most situations (5). The frequency of this interference in IA ranging from 0.4% to 0.5% has been reported in different studies (6,7).

Although IA interference may be seen with different markers, CT interference was reported in only several cases. We present a multiple-IA interference in a patient with falsely elevated CT due to interaction with HAbs.

## CASE

**Figure 1:**
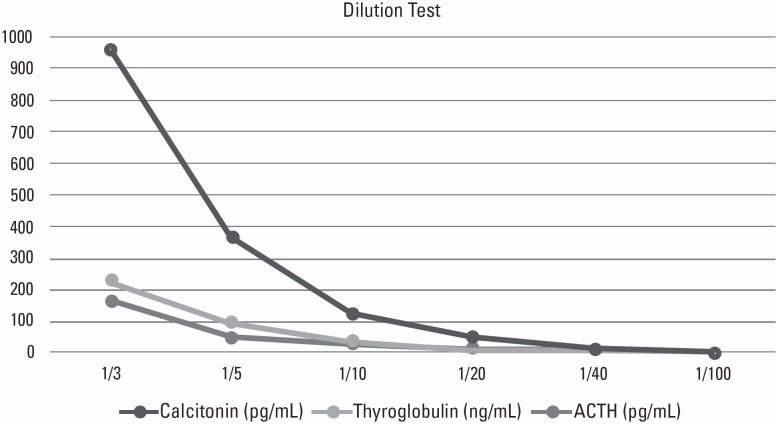
Following serial dilutions at various dilution rates, the levels of calcitonin, thyroglobulin, and ACTH showed non-linearity results that indicate the possibility of immunoassay interference.

A 50-year-old female patient was examined at an outpatient clinic with the complaint of diarrhea that had persisted for one year. She was using levothyroxine for Hashimoto thyroiditis and had bariatric surgery 3 years prior. The patient was evaluated for chronic diarrhea. She was suffering from diarrhea for more than 2 years and did not have any alarming symptoms like fever, weight loss, or bloody diarrhea. She described loose stool 3 times per day, which ceased during fasting or at night. Her thyroid function tests were normal, celiac antibodies were negative, and no parasites were detected. The CT level was screened to exclude hormone-secreting tumors like carcinoid syndrome. She was referred to endocrinology upon measuring her serum CT levels (3199 pg/mL [RR: 0-11,5 pg/mL], chemiluminescent immunometric assay). Its elevation was confirmed by the same laboratory in a second sample (CT: 2177 pg/mL). No thyroid nodules were detected in the physical examination or ultrasonography. Although repeated CT measurements showed high levels, the carcinoembryonic antigen (CEA) level was in the normal range (CEA: 0.44 ng/mL [RR: 0-2,9 ng/mL]). Secondary causes of hypercalcitoninemia, such as renal insufficiency and hypercalcemia, were ruled out. Different markers and hormones were tested to determine if there was an interference in immunometric assay measurements. The Tg and Adrenocorticotropic hormone (ACTH) levels were also found to be elevated (TG: 626 ng/mL [RR; 0.7-84 ng/mL], ACTH: 492 pg/mL [RR: 0-45 pg/mL]). The recovery of the CT concentration after precipitation with polyethylene glycol (PEG) was markedly low indicating the formation of macro-aggregates (pre-PEG CT: 2,574 pg/mL *vs.* post-PEG CT: 43.6 pg/mL). Similar results were also observed with Tg and ACTH when treated with PEG (Table 1). Serial dilutions showed non-linearity in plasma concentrations of ACTH, CT, and Tg (Figure 1). Thus, IA interference was suspected so samples were tested at a different center (Duzen Laboratories, Ankara, Turkey). When CT was evaluated with the same IA platform at a different center, it was found to be similarly elevated. On the other hand, CT, ACTH, and Tg were found to be in the normal range when measured with a different IA platform (Table 1). Additionally, the serum CT measured after pretreatment with heterophilic blocking tube (HBT) was undetectably low (<1 pg/mL) demonstrating the heterophilic antibody interference. Afterward, the patient was referred to the gastroenterology department and planned an endoscopic evaluation for chronic diarrhea.

**Table 1:** Evaluation of immunoassay interference for calcitonin, ACTH, and thyroglobulin measurements

	Before PEG (A)	After PEG (A)	Recovery (%)	B
Calcitonin (pg/mL) (RR)	2574 (0-11.5)	43.6	1.6	0.63 (5.17-9.82)
ACTH (pg/mL) (RR)	492 (0-45)	< 0.5	<0.1	15.6 (7.2-63.3)
Thyroglobulin (ng/mL) (RR)	691 (0.7-84)	13.9	2.0	12.3 (0-85)

Hormone levels were measured before and after the serum was treated with PEG to assess for assay interference. Chemiluminescence and electrochemiluminescence immunoassay methods were used to measure analytes at A and B platforms, respectively.

RR: reference range; PEG: polyethylene glycol; ACTH: adrenocorticotropic hormone.

## METHODS

Screening for IA interference and macrocalcitonin was carried out through the PEG precipitating method, which is usually used to determine macroprolactinemia (8). In brief, serum samples (250 µL) were treated with 250 µL of 25% PEG to precipitate γ-globulin fractions; the vortex was mixed for 1 min and incubated for 10 min at room temperature then centrifuged at 4,000 rpm for 5 min at 25 °C. Hormone levels were measured in the supernatant. Results were multiplied by 2 to correct for dilution. Recovery (%) was calculated by the original hormone value. To rule out any potential interference from HAbs, we also examined the linearity of serum analyte results following serial dilution with an equal amount of distilled water.

The patient sample was treated with specific heterophilic antibodies blocking reagent (HBR, Scantibodies Laboratory Inc., Santee, CA) according to recommended instructions by the manufacturer to detect interference with HAb (9). In brief, 500 μL of patient serum was pipetted into a specific tube containing the blocking reagent. Tube was gently inverted 5 times and incubated for 1 h at 18– 28 °C. HAb lose their ability to interfere with immunoassays once the binders have been attached to them. Afterward, the sample was tested for CT on Immulite 2000 XPi. IA platforms, kits, and methods to measure analytes are summarized in Table 2.

**Table 2:** Antibody types and methods of different immunoassay platforms to measure calcitonin, ACTH, and thyroglobulin

	A (CLIA)	B (ECLIA)
Calcitonin	Monoclonal murine anti-CT ab and polyclonal goat anti-CT ab	Biotinylated monoclonal anti-CT ab (murine) and ruthenium complex labeled monoclonal CT-specific ab (murine)
ACTH	Monoclonal murine anti-ACTH ab and polyclonal rabbit anti-ACTH ab	Biotinylated monoclonal anti-ACTH ab (murine) and ruthenium complex labeled monoclonal ACTH-specific ab (murine)
Thyroglobulin	Anti-Tg murine monoclonal antibody ab and polyclonal sheep anti-thyroglobulin antibody ab	Biotinylated monoclonal anti-Tg ab (murine) and ruthenium complex labeled monoclonal Tg-specific ab (murine)

A platform: The tests were performed on Siemens Immulite 2000 XPi immunoassay analyzer by the chemiluminescence immunoassay (CLIA). B platform: ACTH and calcitonin tests were performed on Roche Cobas e 601, and thyroglobulin test was performed on Roche Cobas e 801 immunoassay analyzers by the electrochemiluminescence immunoassay (ECLIA).

CT: calcitonin; ACTH: adrenocorticotropic hormone; Tg: thyroglobulin.

## DISCUSSION

This is a rare case of multiple IA interference resulting in falsely elevated levels of CT, Tg, and ACTH.

Hormone-secreting tumors like carcinoid syndrome usually cause secretory diarrhea which is associated with large volumes of watery stools and persists during fasting (10). Our patient has no alarming symptoms and diarrhea resolves during night or fasting, unlike secretory diarrhea. Common causes of chronic diarrhea must be excluded before performing additional tests for carcinoid syndrome.

CT concentration was analyzed by non-competitive (sandwich) IA. When interference was suspected it may be possible to interfere with other hormones measured by same the method (4). Other than CT, in our patient, Tg and ACTH were found to be elevated inappropriately. PEG precipitation, dilution, and recovery tests could be useful in detecting this situation (11). Recovery of CT, Tg, and ACTH after being treated with PEG, indicates macro-aggregates in the serum. Also with this result, the finding of nonlinearity on a serial dilution of these hormones suggests the presence of assay interference in our patient.

Alves and cols. reported that falsely elevated CT levels in three patients with medullar thyroid carcinoma may be caused by macrocalcitonin (macro-CT). Although the exact etiology of macro-CT is not found yet, the formation of macroaggregates between the peptide and Ig is the possible main cause. After being treated with PEG, CT levels recovered in macro-CT, but CT results maintained linearity after the dilution test which excludes the presence of HAb unlike our case (12).

Assessing falsely elevated markers with different IA kits is another approach to determine interference (3,13). In this case, the CT was found to be falsely elevated at two different centers, but it later turned out that the two laboratories had used the same IA platform. On the contrary, the Tg, ACTH, and CT were within the normal range once it was assessed with a different kit. IA platforms used in the first center had polyclonal rabbit anti-ACTH, goat anti-CT, and sheep anti-Tg antibodies to determine ACTH, CT, and Tg levels, respectively. On the other hand, murine-based antibodies were used in the kit in which hormones were detected within the normal range (Table 2).

HAbs may bind to capture and trace antibodies, producing an assay interference (14). ACTH interference by HAbs was reported in patients with adrenal incidentaloma (15). To eliminate such interference, our patient's serum was treated with HBT before the assay, which significantly reduced the CT concentration. With this result, as previously reported, a false high CT level was found to be caused by HAb interference (3,14,16,17).

In conclusion, various laboratory markers measured by the immunometric assay may be found to be falsely elevated due to interference with HAbs. This may lead to unnecessary investigations. Thus, when discordant laboratory results are found, IA interference should be suspected.
